# Pericardial Tamponade and Other Cardiac Complications in POEMS Syndrome

**DOI:** 10.1016/j.jaccas.2020.12.027

**Published:** 2021-02-17

**Authors:** Jacqueline Levene, Nathanial Murray, Sima Desai, Timothy F. Simpson, Chafic Karam, Rebecca Silbermann, Ahmad Masri

**Affiliations:** a Department of Medicine, Oregon Health & Science University, Portland, Oregon, USA; b Knight Cardiovascular Institute, Oregon Health & Science University, Portland, Oregon, USA; c Department of Neurology, Oregon Health & Science University, Portland, Oregon, USA; d Amylodosis Center, Oregon Health & Science University, Portland, Oregon, USA; e Department of Hematology and Oncology, Oregon Health & Science University, Portland, Oregon, USA

**Keywords:** cardiomyopathy, pericardial effusion, tamponade, LV, left ventricle, POEMS, polyneuropathy, organomegaly, endocrinopathy, monoclonal gammopathy, and skin changes, RV, right ventricle, TTE, transthoracic echocardiogram, VEGF, vascular endothelial growth factor

## Abstract

Polyneuropathy, organomegaly, endocrinopathy, monoclonal gammopathy, and skin changes (POEMS) is a multiorgan syndrome with rare and heterogenous cardiac manifestations. We present the case of a man with pericardial effusion complicated by cardiac tamponade, new onset atrial fibrillation, and high-degree atrioventricular block leading to a diagnosis of POEMS syndrome. (**Level of Difficulty: Advanced.**)

## Graphical abstract

**Figure:**
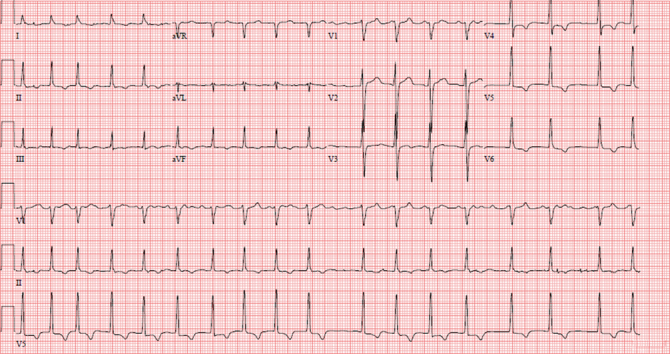

A 66-year-old man with hypothyroidism and demyelinating polyneuropathy presented to the emergency department with new-onset atrial fibrillation with rapid ventricular rates up to 160 beats/min (Figure 1). Two weeks prior, he was admitted to an outside hospital with pleuritic, substernal chest pain associated with dyspnea and was found to have a small circumferential pericardial effusion on echocardiogram (Figure 2, Video 1). He was started on colchicine and ibuprofen for presumed idiopathic pericarditis and discharged home.

**Learning Objectives**

• To recognize the cardiac manifestations of POEMS syndrome and the role of VEGF assay.
• To review the diagnostic findings of multimodality cardiac imaging in POEMS syndrome.

**Figure 1:**
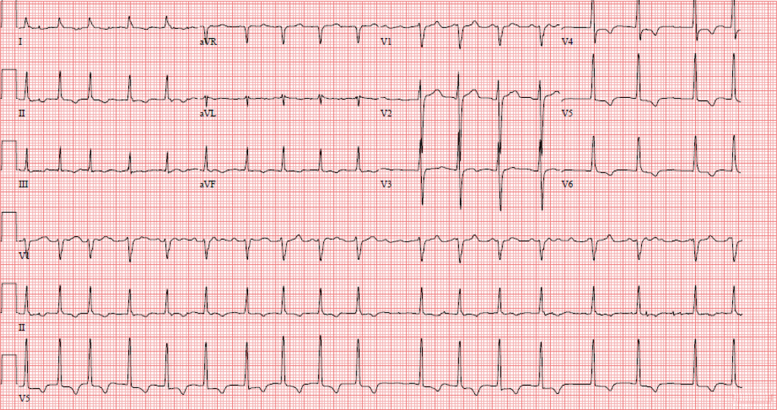
Electrocardiogram at Presentation Showing Atrial Fibrillation and Lateral T-Wave Inversions (V_4_ to V_6_)

**Figure 2:**
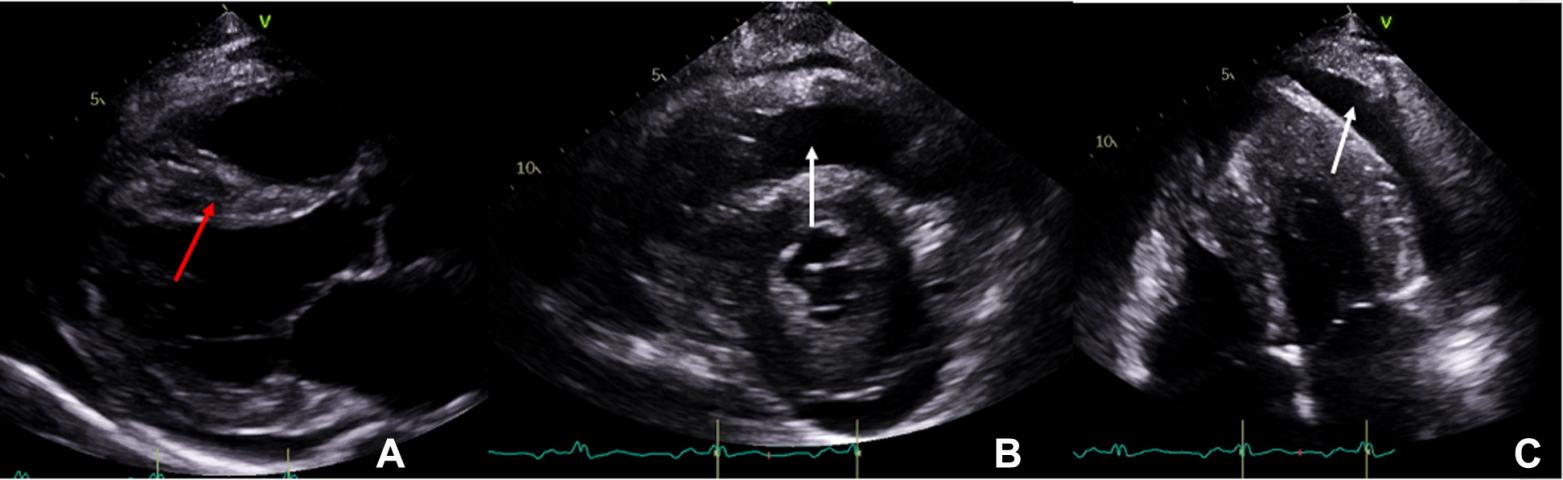
Initial Transthoracic Echocardiogram Initial transthoracic echocardiogram demonstrating a small pericardial effusion, 1.8 cm in maximum diameter next to the lateral left ventricle wall but 1.2 cm elsewhere (white arrows) and concentric mild left ventricular hypertrophy toward the mid-ventricle and apex (red arrow, foreshortened windows). **(A)** Parasternal long-axis view. **(B)** Parasternal short-axis view. **(C)** Apical 4-chamber view.

Upon representation, the patient was afebrile and his blood pressure was 120/74 mm Hg, heart rate ranged from 103 to 160 beats/min, respirations were 16 breaths/min, and Spo_2_ was 100% on 1-l nasal cannula. He appeared cachectic with bitemporal wasting, and rales were heard in bilateral lung bases. Cardiovascular examination demonstrated irregularly irregular tachycardia, a pronounced P2, and a 2/6 holosystolic murmur loudest at the apex. Jugular venous pressure was 15 cm H_2_O. Kussmaul’s sign was not present. Extremities were warm, with 2+ pitting edema. There was loss of bilateral upper and lower extremity deep tendon reflexes and sensation to the midcalf, with preserved muscle strength. His skin appeared hyperpigmented.

An urgent transthoracic echocardiogram (TTE) showed a large pericardial effusion measuring 4.2 cm in its maximum dimension and echocardiographic signs of tamponade physiology (Figure 3, Video 2) for which an emergent pericardiocentesis was performed. After pericardiocentesis, his heart rate improved to 120 beats/min.

**Figure 3:**
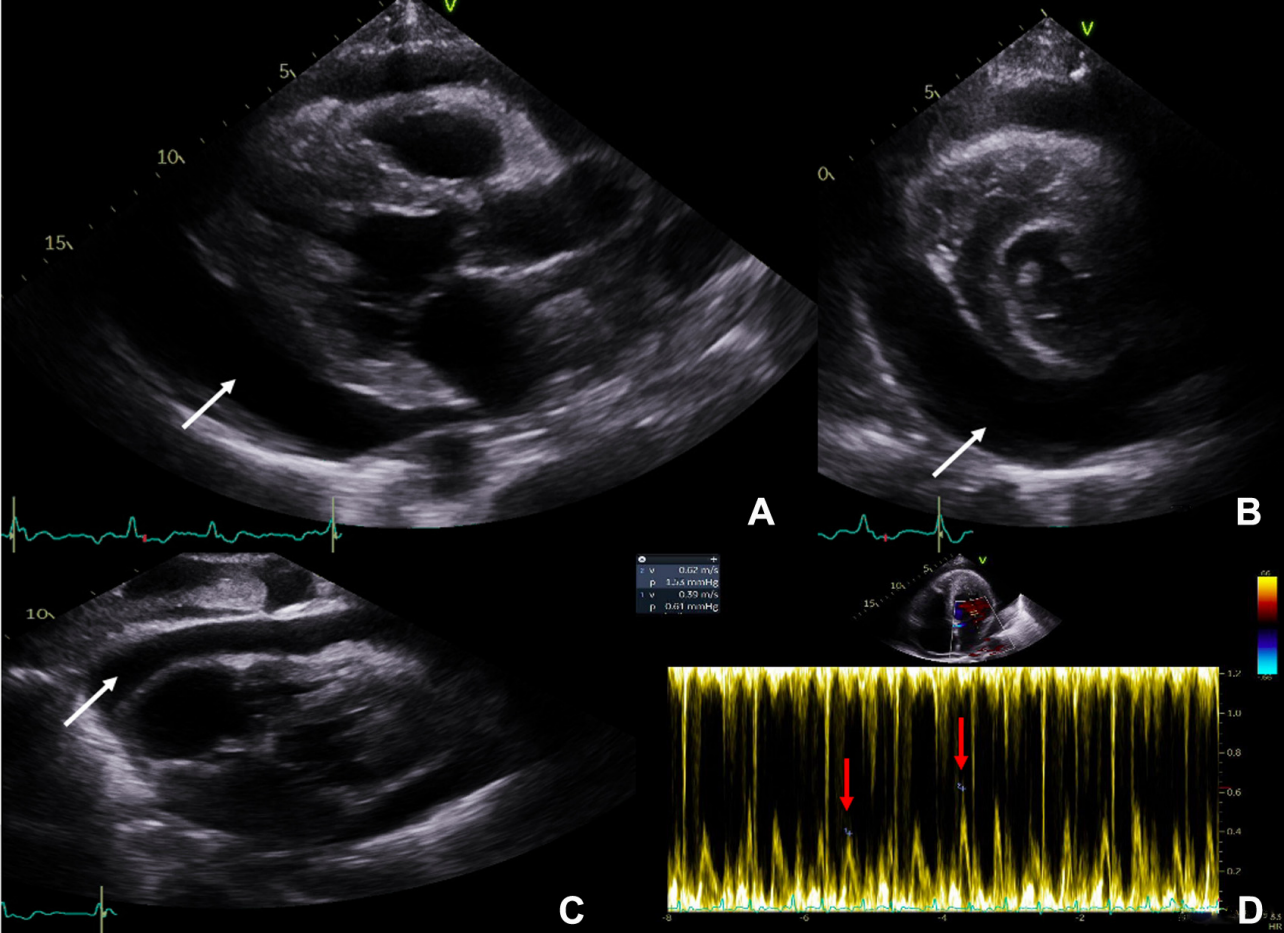
Subsequent Urgent Transthoracic Echocardiogram Urgent limited transthoracic echocardiogram with a large pericardial effusion **(white arrows)** with right ventricular diastolic collapse and respiratory flow variation across the mitral valves (**red arrows**, variation of 37%; a respirometer was not used in the emergency room settings). **(A)** Parasternal long-axis view. **(B)** Parasternal short-axis view. **(C)** Subcostal views. **(D)** Transmitral flow variation during respiration.

Pericardial fluid analysis demonstrated a transudative inflammatory effusion and negative culture results. Laboratory analysis was notable for a white blood cell count of 13,000/mm^3^, creatinine level 1.68 mg/dl, C-reactive protein level 90 mg/l, and erythrocyte sedimentation rate of 44 mm/h. N-terminal pro-brain natriuretic peptide level was 9,947 pg/ml, and troponin I was undetectable. A chest radiograph demonstrated a mildly enlarged cardiac silhouette, small bilateral pleural effusions, and a single sclerotic T6 vertebral body. The patient continued to have atrial fibrillation with rapid ventricular rate and subsequently developed sinus arrest with slow junctional escape requiring placement of a temporary transvenous pacer.

## Medical History

The patient had a history of hypothyroidism and 4 years of progressive weakness and numbness in the feet contributed to Charcot-Marie-Tooth disease. Recent thoracic spine magnetic resonance imaging done for evaluation of his progressive weakness revealed a T6 sclerotic vertebra. The patient had no personal or family cardiac history.

## Differential Diagnosis

It was difficult initially to reconcile such profound cardiac disease with the patient’s systemic symptoms in a unifying diagnosis. Idiopathic pericarditis in isolation or as a part of a serositis syndrome were at the top of the differential diagnosis. Autoimmune (e.g., lupus, rheumatoid arthritis, adult Still’s disease), infectious, and malignant etiologies were also considered. Light-chain amyloidosis was a particular consideration, given his conduction disturbances with arrhythmia and left ventricular hypertrophy. However, the presence of anasarca, neuropathy, skin changes, and a sclerotic bone lesion raised suspicion for polyneuropathy, organomegaly, endocrinopathy, monoclonal gammopathy, and skin changes (POEMS) syndrome.

## Investigations

A repeat TTE demonstrated a small pericardial effusion, a mildly enlarged left ventricle (LV) with mild concentric left ventricular hypertrophy, mildly reduced systolic function with a left ventricular ejection of 52%, mildly reduced right ventricular (RV) systolic function, right ventricular systolic pressure of 71 mm Hg, and severe biatrial enlargement (Video 3). A thoracentesis showed an exudative effusion with negative culture results and cytology without evidence of malignancy. Test results for autoantibodies for common rheumatologic diseases were negative. Serum quantitative immunoglobulins were normal, and serum free light chains were symmetrically elevated (kappa: 49.24 mg/l; lambda: 61.99 mg/l; ratio: 0.79). Serum protein electrophoresis demonstrated an abnormal trace immunoglobulin A lambda monoclonal protein. Urine studies demonstrated monoclonal free lambda light chains (21.2 mg/24 h). Peripheral blood flow cytometry was unremarkable. Vascular endothelial growth factor (VEGF) levels were 575 pg/ml (reference: <96.2 pg/ml). Bone marrow biopsy demonstrated a normocellular marrow with trilineage hematopoiesis, lymphoid aggregates of small T and B cells, and a small polyclonal plasma cell population. Bone marrow stains and serum testing results for human herpesvirus 8 was negative. The fat pad biopsy result was negative for amyloid. Computed tomography scan demonstrated hepatosplenomegaly and numerous subcentimeter sclerotic lesions throughout the axial and appendicular skeleton. Free testosterone level was 1 pg/ml (reference: 47 to 244 pg/ml) and follicle-stimulating hormone and luteinizing hormone were both elevated at 26 and 16 mIU/ml, respectively.

## Management

The size of the pericardial effusion remained stable. Conduction disease, intermittent sinus arrest with slow junctional escape, and paroxysmal atrial fibrillation continued to occur, necessitating a permanent dual-chamber pacemaker. For POEMS syndrome associated with numerous bone lesions, the patient was started on cyclophosphamide, carfilzomib, and dexamethasone.

## Discussion

Cardiac manifestations of POEMS syndrome are rare, yet our patient’s cardiac complications are what led to the diagnosis of POEMS syndrome (1). Our patient was diagnosed with POEMS syndrome after meeting the major criteria of polyneuropathy and monoclonal gammopathy and minor criteria of organomegaly, extravascular volume overload, endocrinopathy, and skin changes. The aforementioned findings are typical for POEMS syndrome; however, the diagnosis is often difficult to make (2).

Multisystemic end-organ effects are in part mediated through elevated cytokines, immunoglobulins, and growth factors, most notably VEGF, leading to microvascular hyperpermeability, extravasation of fibrinogen, and myocardial fibrosis (3). The cardiac manifestations in POEMS syndrome are rare and heterogenous: cardiomyopathy, pericarditis, and pericardial effusions have all been described previously, and heart failure in particular has been cited as a cause of death in up to a third of patients (4,5). Pericarditis is rarely described, particularly when combined with tamponade and arrhythmias, and there are few cases reporting conduction disturbances or arrythmia (6, 7, 8). Pulmonary hypertension with resultant right ventricular dysfunction is more commonly reported (9). Extravascular fluid accumulation likely results from VEGF-mediated vascular permeability (8). The majority of endomyocardial biopsy specimens in POEMS syndrome do not stain for VEGF or other specific proteins but rather demonstrate hypertrophy and fibrosis, further supporting a paraneoplastic process rather than an infiltrative process driving disease sequelae (1,3).

There are no specific therapies for the cardiac manifestations of POEMS, although regression of cardiac dysfunction has been observed with treatment of the underlying plasma cell disorder (3). When a single lesion (plasmacytoma) exists, radiation therapy may be considered, whereas systemic chemotherapy is pursued when diffuse bone marrow involvement is present (10).

## Follow-Up

Our patient was started on systemic chemotherapy with improvement of his dyspnea, neuropathy, and peripheral edema. Repeat TTE 1 month later demonstrated improved LV and RV systolic function, a decrease in RV systolic pressure, and interval improvement of pericardial and pleural effusions. His VEGF level decreased to 263 pg/ml, free light chains normalized, and the serum monoclonal protein is no longer detectable.

## Conclusions

This case demonstrates the importance of recognizing cardiovascular manifestations as the initial or cardinal presentation of an underlying systemic disease.

## Funding Support and Author Disclosures

Dr. Karam has received personal compensation for consulting, serving on the Scientific Advisory Board, speaking, or performing other activities with Akcea, Alnyalm, Argenx, Biogen, CSL Behring, and Sanofi Genzyme; and has received research support from Akcea and Sanofi Genzyme. Dr. Silbermann has served on advisory boards for Janssen, Karyopharm, and Sanofi. Dr. Masri has received research grants from Pfizer, Akcea, and Ultromics (paid to Oregon Health & Science University); and has received consulting fees from Eidos, Ionis, Alnylam, and Cytokinetics. All other authors have reported that they have no relationships relevant to the contents of this paper to disclose.

## Appendix

Supplemental Video 1 Initial transthoracic echocardiogram demonstrating a small pericardial effusion, 1.8 cm in maximum diameter next to the lateral LV wall, but 1.2 cm elsewhere (white arrows), and concentric mild left ventricular hypertrophy towards the mid ventricle and apex (red arrow, foreshortened windows). (A) parasternal long axis view (B) parasternal short axis view and (C) apical four-chamber view.

Supplemental Video 2 Urgent limited echo with a large pericardial effusion (white arrows) with RV diastolic collapse and respiratory flow variation across the mitral valves (red arrows, variation of 37%, a respirometer was not utilized in the emergency room settings). (A) parasternal long axis view (B) parasternal short axis view (C) subcostal views and (D) trans-mitral flow variation during respiration.

Supplemental Video 3 Repeat TTE following pericardiocentesis showed a small effusion, EF of 52%, mildly reduced RV systolic function and severe bi-atrial enlargement.
